# Supplementary figures and images for: Reduction of oxidative stress on DNA and RNA in obese patients after Roux-en-Y gastric bypass surgery—An observational cohort study of changes in urinary markers
Source: PLoS One. 2020 Dec 14;15(12):e0243918. doi: 10.1371/journal.pone.0243918 (PMC7735613; doi:10.1371/journal.pone.0243918)

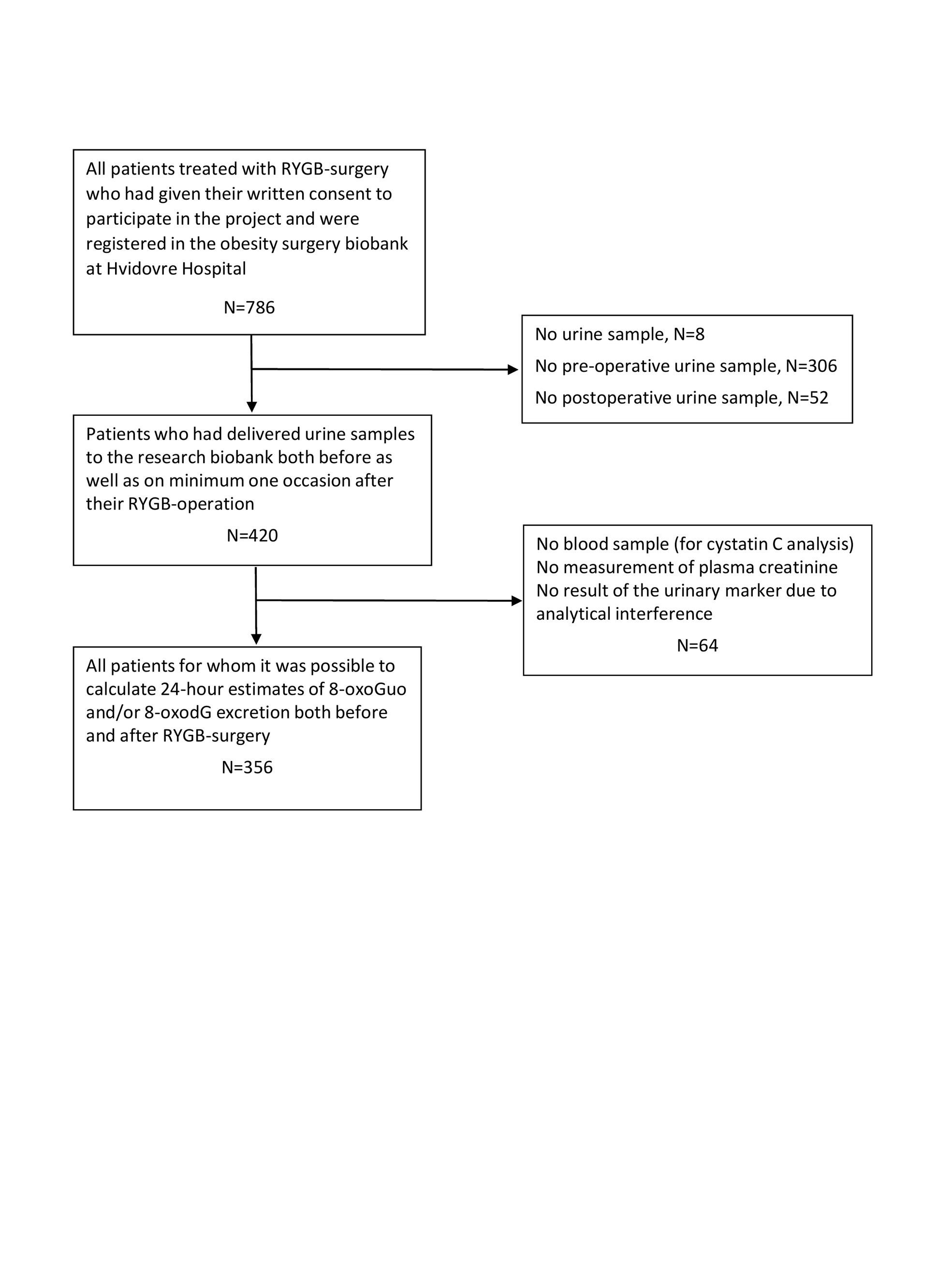

Supplement: S1 Fig — (TIF) [file pone.0243918.s001.tif]

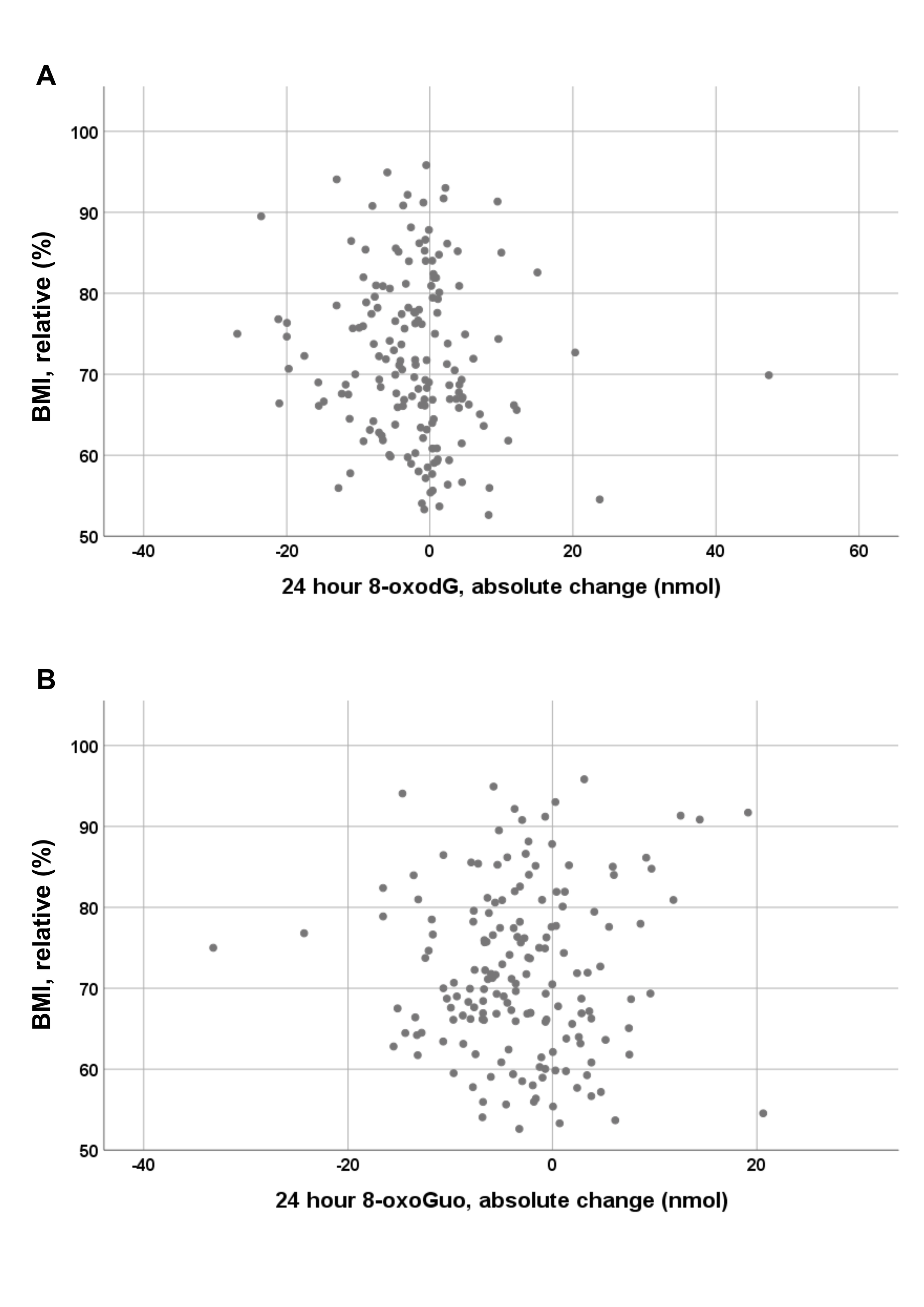

Supplement: S2 Fig — The graph is a x-y plot, where delta-values of 8-oxodG (A) and 8-oxoGuo (B) are plotted against the relative BMI for individual patients, 24 months after RYGB. On the x-axis, 0 nmol represents no change in urinary excretion of the marker. On the y-axis, 100% represents the preoperative BMI-value. (TIF) [file pone.0243918.s002.tif]
