# Supplementary material for: Reduction of oxidative stress on DNA and RNA in obese patients after Roux-en-Y gastric bypass surgery—An observational cohort study of changes in urinary markers
Source: PLoS One. 2020 Dec 14;15(12):e0243918. doi: 10.1371/journal.pone.0243918 (PMC7735613; doi:10.1371/journal.pone.0243918)
Supplement: S1 Table — Data are reported as Mean (SD), except for the parameters reported as a percentage or number. Age is on the day of Roux-en-Y gastric bypass (RYGB) surgery. Clinical data represent the closest available before surgery. NDH, patients with biochemical glucose markers below diagnostic threshold for diabetes and not on antidiabetic treatment; DH, patients with biochemically confirmed diabetes; DH-NDH, patients who obtained remission of diabetes after RYGB; DH-DH, patients who did not obtain remission in diabetes after RYGB; SU, Sulfonylurea; GLP-1, Glucagon-like peptide-1 analogue; DPP4, Dipeptidyl peptidase-4 inhibitor. *All patients also include patients for whom we were not able to confirm diabetes status. †All patients with diabetes also include patients without hyperglycemia after RYGB, but who continued antidiabetic medicine. ‡Apart from the HOMA-IR score and eGFR, biochemical variables are plasma or blood concentrations, unless stated otherwise with U, for urinary. Insulin, C-peptide and Glucose are fasting values. Exact p-values from the one-way ANOVA, followed by a Tukey post hoc test, are reported down to 0.001. Below 0.001, P-values are reported as < 0.001 or < 0.0001. A Welch—Satterthwaite correction followed by a Games—Howell post hoc test have been used as appropriate, to adjust for unequal variances and for HbA1c, glucose and triglycerides, significant differences were confirmed with a Kruskal-Wallis H-test, as these variables did not have a normal distribution. (DOCX) [file pone.0243918.s003.docx]

| **S1 Table. Preoperative clinical and laboratory data for all Roux-en-Y Gastric Bypass-operated patients and patients divided in subgroups according to diabetes status.** | | | | | | | | | | | | |
| --- | --- | --- | --- | --- | --- | --- | --- | --- | --- | --- | --- | --- |
|  | **All patients (n=356)*** | | **NDH, Patients without diabetes (n=241)** | | **DH, Patients with diabetes (n=96)** | | | | | | ***P*** | |
|  |  |  |  |  | All (n=96)† | | DH-NDH (n=61) | | DH-DH (n=31) | | NDH vs. DH | DH-NDH vs. DH-DH |
|  | n | Mean (SD) | n | Mean (SD) | n | Mean (SD) | n | Mean (SD) | n | Mean (SD) |  |  |
| **Clinical data** |  |  |  |  |  |  |  |  |  |  |  |  |
| Age (years) | 356 | 44.2 (9.6) | 241 | 41.7 (9.1) | 96 | 49.8 (8.3) | 61 | 49.1 (9.1) | 31 | 50.8 (6.9) | < 0.0001 | 0.683 |
| Females (%) | 356 | 69.1 | 241 | 75.5 | 96 | 54.0 | 61 | 54.0 | 31 | 55.0 | < 0.001 | 0.998 |
| Height (cm) | 356 | 171.3 (9.5) | 241 | 170.7 (9.5) | 96 | 172.1 (9.5) | 61 | 173.3 (8.6) | 31 | 169.9 (11.3) | 0.245 | 0.251 |
| Bodyweight (kg) | 356 | 124.0 (21.9) | 241 | 124.9 (22.5) | 96 | 122.2 (21.7) | 61 | 125.9 (22.6) | 31 | 115.8 (17.6) | 0.318 | 0.096 |
| BMI (kg/m^2^) | 356 | 42.1 (5.6) | 241 | 42.7 (5.6) | 96 | 41.2 (5.6) | 61 | 41.8 (5.9) | 31 | 40.0 (3.9) | 0.023 | 0.218 |
| Body Surface Area (m^2^) | 356 | 2.32 (0.24) | 241 | 2.32 (0.25) | 96 | 2.31 (0.24) | 61 | 2.35 (0.24) | 31 | 2.24 (0.23) | 0.776 | 0.104 |
| Blood Pressure: |  |  |  |  |  |  |  |  |  |  |  |  |
| Systolic (mmHg) | 353 | 127.7 (14.2) | 238 | 126.9 (14.3) | 96 | 128.8 (13.7) | 61 | 128.7 (13.8) | 31 | 128.2 (13.9) | 0.271 | 0.984 |
| Diastolic (mmHg) | 353 | 81.6 (9.6) | 238 | 81.4 (10.1) | 96 | 81.5 (8.7) | 61 | 80.9 (7.1) | 31 | 81.7 (10.2) | 0.976 | 0.686 |
| Use of tobacco: |  |  |  |  |  |  |  |  |  |  |  |  |
| Present/Previous/Never (n) | 355 | 61/110/184 | 240 | 42/67/131 | 96 | 17/41/38 | 61 | 14/22/25 | 31 | 2/17/12 |  |  |
| Present smoker (%) |  | 17.2 |  | 17.5 |  | 17.7 |  | 22.9 |  | 6.5 | 0.964 | 0.070 |
| Lipid-lowering treatment (%) | 356 | 26.0 | 241 | 10.0 | 96 | 61.0 | 61 | 51.0 | 31 | 77.0 | < 0.0001 | 0.026 |
| Anti-hypertensive treatment (%) | 356 | 44.0 | 241 | 32.0 | 96 | 75.0 | 61 | 69.0 | 31 | 84.0 | < 0.0001 | 0.223 |
| Anti-diabetic treatment: |  |  |  |  |  |  |  |  |  |  |  |  |
| Metformin/Insulin/SU/GLP-1/DPP4 (n) |  |  |  |  |  |  | 61 | 38/9/7/14/4 | 31 | 26/14/7/15/4 |  |  |
| Metformin (%) |  |  |  |  |  |  |  | 62.3 |  | 83.9 |  | 0.055 |
| **Laboratory data‡** |  |  |  |  |  |  |  |  |  |  |  |  |
| HbA_1c_ (mmol/mol) | 348 | 39.1 (10.1) | 235 | 34.4 (3.7) | 94 | 51.2 (11.8) | 60 | 48.7 (11.2) | 30 | 57.5 (11.1) | < 0.0001 | 0.002 |
| HbA_1c_ (%) |  | 5.7 (0.92) |  | 5.3 (0.34) |  | 6.8 (1.08) |  | 6.6 (1.02) |  | 7.4 (1.02) |  |  |
| Insulin (pmol/L) | 346 | 125.2 (102.4) | 235 | 115.0 (88.8) | 92 | 144.3 (96.8) | 60 | 150.9 (80.9) | 29 | 128.9 (125.3) | 0.009 | 0.534 |
| C-peptide (pmol/L) | 349 | 1241.4 (496.9) | 237 | 1180 (459.0) | 93 | 1371.5 (536.3) | 60 | 1466.1 (501.9) | 30 | 1150.7 (553.7) | 0.003 | 0.009 |
| P-Glucose (mmol/L) | 351 | 6.3 (2.2) | 238 | 5.4 (0.6) | 96 | 8.4 (3.3) | 61 | 7.9 (2.5) | 31 | 9.8 (4.1) | < 0.0001 | 0.049 |
| HOMA2-IR | 336 | 3.0 (1.5) | 228 | 2.7 (1.1) | 91 | 3.7 (2.0) | 60 | 3.7 (1.4) | 28 | 3.6 (3.0) | < 0.0001 | 0.966 |
| Cholesterol: |  |  |  |  |  |  |  |  |  |  |  |  |
| Total (mmol/L) | 354 | 4.76 (1.03) | 239 | 4.96 (0.94) | 96 | 4.35 (1.16) | 61 | 4.34 (0.96) | 31 | 4.47 (1.53) | < 0.0001 | 0.902 |
| HDL- (mmol/L) | 354 | 1.15 (0.29) | 239 | 1.18 (0.28) | 96 | 1.07 (0.33) | 61 | 1.06 (0.32) | 31 | 1.12 (0.37) | 0.002 | 0.656 |
| LDL- (mmol/L) | 349 | 2.87 (0.94) | 236 | 3.08 (0.86) | 94 | 2.41 (1.01) | 59 | 2.46 (0.82) | 31 | 2.40 (1.35) | < 0.0001 | 0.973 |
| VLDL- (mmol/L) | 348 | 0.73 (0.34) | 236 | 0.68 (0.30) | 93 | 0.85 (0.38) | 58 | 0.78 (0.31) | 31 | 0.95 (0.49) | < 0.001 | 0.198 |
| Triglycerides (mmol/L) | 354 | 1.70 (1.01) | 239 | 1.56 (0.80) | 96 | 2.01 (1.39) | 61 | 2.01 (1.53) | 31 | 2.15 (1.18) | < 0.001 | 0.886 |
| Creatinine (µmol/L) | 356 | 67.3 (14.0) | 241 | 67.4 (11.8) | 96 | 68.0 (18.4) | 61 | 66.8 (14.7) | 31 | 68.3 (23.7) | 0.775 | 0.944 |
| Cystatin C (mg/L) | 356 | 0.90 (0.19) | 241 | 0.90 (0.16) | 96 | 0.92 (0.25) | 61 | 0.92 (0.23) | 31 | 0.89 (0.24) | 0.380 | 0.773 |
| eGFR Creatinine (mL/(min x 1.73m^2^)) | 340 | 84.7 (8.8) | 235 | 84.7 (8.1) | 86 | 84.3 (10.6) | 53 | 85.3 (8.6) | 29 | 83.8 (8.7) | 0,752 | 0.729 |
| eGFR Cystatin C (mL/(min x 1.73m2)) | 356 | 92.4 (19.4) | 241 | 92.7 (18.3) | 96 | 90.4 (22.1) | 61 | 90.1 (21.3) | 31 | 93.6 (22.1) | 0.340 | 0.687 |
| U-Creatinine (mmol/L) | 350 | 15.3 (7.7) | 237 | 15.5 (7.3) | 95 | 15.1 (8.9) | 60 | 15.6 (8.6) | 31 | 14.4 (9.8) | 0.725 | 0.789 |

Data are reported as Mean (SD), except for the parameters reported as a percentage or number. Age is on the day of Roux-en-Y gastric bypass (RYGB) surgery. Clinical data represent the closest available before surgery. NDH, patients with biochemical glucose markers below diagnostic threshold for diabetes and not on antidiabetic treatment; DH, patients with biochemically confirmed diabetes; DH-NDH, patients who obtained remission of diabetes after RYGB; DH-DH, patients who did not obtain remission in diabetes after RYGB; SU, Sulfonylurea; GLP-1, Glucagon-like peptide-1 analogue; DPP4, Dipeptidyl peptidase-4 inhibitor. *All patients also include patients for whom we were not able to confirm diabetes status. †All patients with diabetes also include patients without hyperglycemia after RYGB, but who continued antidiabetic medicine. ‡Apart from the HOMA-IR score and eGFR, biochemical variables are plasma or blood concentrations, unless stated otherwise with U, for urinary. Insulin, C-peptide and Glucose are fasting values. Exact *p*-values from the one-way ANOVA, followed by a Tukey post hoc test, are reported down to 0.001. Below 0.001, *P*-values are reported as < 0.001 or < 0.0001. A Welch–Satterthwaite correction followed by a Games–Howell post hoc test have been used as appropriate, to adjust for unequal variances and for HbA1c, glucose and triglycerides, significant differences were confirmed with a Kruskal-Wallis H-test, as these variables did not have a normal distribution.
