# Supplementary material for: Reduction of oxidative stress on DNA and RNA in obese patients after Roux-en-Y gastric bypass surgery—An observational cohort study of changes in urinary markers
Source: PLoS One. 2020 Dec 14;15(12):e0243918. doi: 10.1371/journal.pone.0243918 (PMC7735613; doi:10.1371/journal.pone.0243918)
Supplement: S2 Table — Data are reported as mean with a 95% confidence interval. NDH, patients with biochemical glucose markers below diagnostic threshold for diabetes and not on antidiabetic treatment; DH, patients with biochemically confirmed diabetes. All patients also include patients for whom we were not able to confirm diabetes status. Exact P-values from unpaired t-tests are reported down to 0.001. (DOCX) [file pone.0243918.s004.docx]

| **preoperative 8-oxodG (nmol/mmol creatinine)** | | | | | | | |
| --- | --- | --- | --- | --- | --- | --- | --- |
|  | all | | NDH | | DH | | DH vs. NDH |
|  | n | mean (95 % CI) | n | mean (95 % CI) | n | mean (95 % CI) | *P* |
| All | 356 | 1.64 (1.57 – 1.70) | 241 | 1.65 (1.56 - 1.73) | 96 | 1.64 (1.51 - 1.76) | 0.911 |
| Females | 246 | 1.65 (1.57 - 1.74) | 182 | 1.66 (1.56 - 1.76) | 52 | 1.62 (1.44 - 1.80) | 0.716 |
| Males | 110 | 1.60 (1.49 - 1.71) | 59 | 1.60 (1.45 - 1.75) | 44 | 1.65 (1.47 - 1.83) | 0.643 |
| F vs. M | *P* | 0.455 |  | 0.535 |  | 0.804 |  |
| **preoperative 8-oxoGuo (nmol/mmol creatinine)** | | | | | | | |
|  | all | | NDH | | DH | | DH vs. NDH |
|  | n | mean (95 % CI) | n | mean (95 % CI) | n | mean (95 % CI) | *P* |
| All | 356 | 2.12 (2.06 – 2.18) | 241 | 2.04 (1.97 - 2.11) | 96 | 2.34 (2.20 - 2.48) | < 0.001 |
| Females | 246 | 2.08 (2.00 – 2.15) | 182 | 2.00 (1.92 - 2.09) | 52 | 2.32 (2.12 - 2.53) | 0.005 |
| Males | 110 | 2.21 (2.06 – 2.18) | 59 | 2.15 (2.01 - 2.29) | 44 | 2.36 (2.16 - 2.56) | 0.073 |
| F vs. M | *P* | 0.047 |  | 0.083 |  | 0.802 |  |

**S2 Table. Preoperative 8-oxodG and 8-oxoGuo, normalized to urinary creatinine.**

Data are reported as mean with a 95 % confidence interval. NDH, patients with biochemical glucose markers below diagnostic threshold for diabetes and not on antidiabetic treatment; DH, patients with biochemically confirmed diabetes. All patients also include patients for whom we were not able to confirm diabetes status. Exact *P*-values from unpaired t-tests are reported down to 0.001.
