# Supplementary material for: Reduction of oxidative stress on DNA and RNA in obese patients after Roux-en-Y gastric bypass surgery—An observational cohort study of changes in urinary markers
Source: PLoS One. 2020 Dec 14;15(12):e0243918. doi: 10.1371/journal.pone.0243918 (PMC7735613; doi:10.1371/journal.pone.0243918)
Supplement: Attachment — Submitted filename: Responce to Reviewers.docx [file pone.0243918.s005.docx]

Responce to Reviewers

**PONE-D-20-11081
Reduction of oxidative stress on DNA and RNA in obese patients after Roux-en-Y gastric bypass surgery – an observational cohort study of changes in urinary markers
PLOS ONE

Dear Dr. Carlsson,

Thank you for submitting your manuscript to PLOS ONE, which has now been thoroughly reviewed by three reviewers. After careful consideration, we feel that it has merit but does not fully meet PLOS ONE’s publication criteria as it currently stands. Therefore, we invite you to submit a revised version of the manuscript that addresses the points raised during the review process. Please submit along with the revised version of your manuscript a detailed point-by-point response to all concerns and comments. 

Please submit your revised manuscript by Jul 24 2020 11:59PM. If you will need more time than this to complete your revisions, please reply to this message or contact the journal office at** [**plosone@plos.org**](mailto:plosone@plos.org)**. When you're ready to submit your revision, log on to** [**https://www.editorialmanager.com/pone/**](https://www.editorialmanager.com/pone/) **and select the 'Submissions Needing Revision' folder to locate your manuscript file.

Please include the following items when submitting your revised manuscript:**

- **A rebuttal letter that responds to each point raised by the academic editor and reviewer(s). You should upload this letter as a separate file labeled 'Response to Reviewers'.**
- **A marked-up copy of your manuscript that highlights changes made to the original version. You should upload this as a separate file labeled 'Revised Manuscript with Track Changes'.**
- **An unmarked version of your revised paper without tracked changes. You should upload this as a separate file labeled 'Manuscript'.**

**If you would like to make changes to your financial disclosure, please include your updated statement in your cover letter. Guidelines for resubmitting your figure files are available below the reviewer comments at the end of this letter.

If applicable, we recommend that you deposit your laboratory protocols in protocols.io to enhance the reproducibility of your results. Protocols.io assigns your protocol its own identifier (DOI) so that it can be cited independently in the future. For instructions see:** [**http://journals.plos.org/plosone/s/submission-guidelines#loc-laboratory-protocols**](http://journals.plos.org/plosone/s/submission-guidelines#loc-laboratory-protocols) **We look forward to receiving your revised manuscript.

Kind regards,

Yvonne Böttcher, Ph.D.
Academic Editor
PLOS ONE**

**Journal Requirements:**

**When submitting your revision, we need you to address these additional requirements.**

**1. Please ensure that your manuscript meets PLOS ONE's style requirements, including those for file naming. The PLOS ONE style templates can be found at**

[**https://journals.plos.org/plosone/s/file?id=wjVg/PLOSOne_formatting_sample_main_body.pdf**](https://journals.plos.org/plosone/s/file?id=wjVg/PLOSOne_formatting_sample_main_body.pdf) **and**

[**https://journals.plos.org/plosone/s/file?id=ba62/PLOSOne_formatting_sample_title_authors_affiliations.pdf**](https://journals.plos.org/plosone/s/file?id=ba62/PLOSOne_formatting_sample_title_authors_affiliations.pdf)

Thank you, formats have been adjusted to meet PLOS ONE’s style requirements.

**2. Please provide a sample size and power calculation in the Methods, or discuss the reasons for not performing one before study initiation.**

To our minds, power calculation is a planning tool for controlled trials, and we did not perform such planning calculations as the number of persons in the research cohort was given at the time for initiating this study on collected biobank samples. Instead, we evaluated more grossly if it was worth performing the study. Based on previous power calculations from controlled trials, for example Rasmussen ST et al. Redox Biology 9(2016) 32-38, the power calculation using the same measurements as in the present study indicated 2 x 20 persons to reveal a 20% difference. In the present study the number is much larger, so our pre-trial evaluation was that we would be able to detect clinically relevant differences and, also, with reasonable certainty state that major differences did not exists. Post hoc, we have made a few confirmatory calculations.

To the manuscript, we have added the following lines in Methods section, Data analysis and statistics:

“Based on previous controlled trials with power calculations (43) we evaluated that the study could be conducted with high power and thus acceptable statistical certainty. All differences, both within the same group (comparisons of pre- and postoperative levels) and between groups have been calculated post hoc to have a power above 0.9 when effect-sizes exceeds 0.8.”

**3. We note that you have indicated that data from this study are available upon request. PLOS only allows data to be available upon request if there are legal or ethical restrictions on sharing data publicly. For information on unacceptable data access restrictions, please see** [**http://journals.plos.org/plosone/s/data-availability#loc-unacceptable-data-access-restrictions**](http://journals.plos.org/plosone/s/data-availability#loc-unacceptable-data-access-restrictions)**.**

**In your revised cover letter, please address the following prompts:**

**a) If there are ethical or legal restrictions on sharing a de-identified data set, please explain them in detail (e.g., data contain potentially identifying or sensitive patient information) and who has imposed them (e.g., an ethics committee). Please also provide contact information for a data access committee, ethics committee, or other institutional body to which data requests may be sent.**

**b) If there are no restrictions, please upload the minimal anonymized data set necessary to replicate your study findings as either Supporting Information files or to a stable, public repository and provide us with the relevant URLs, DOIs, or accession numbers. Please see** [**http://www.bmj.com/content/340/bmj.c181.long**](http://www.bmj.com/content/340/bmj.c181.long) **for guidelines on how to de-identify and prepare clinical data for publication. For a list of acceptable repositories, please see** [**http://journals.plos.org/plosone/s/data-availability#loc-recommended-repositories**](http://journals.plos.org/plosone/s/data-availability#loc-recommended-repositories)**.**

**We will update your Data Availability statement on your behalf to reflect the information you provide.**

Thank you for clarifying this, we have addressed the matter in the revised cover letter and have provided contact information to an institutional body to which data requests may be sent.

***************************************************************************************

**Reviewers' comments:

Reviewer's Responses to Questions

Comments to the Author

1. Is the manuscript technically sound, and do the data support the conclusions?

The manuscript must describe a technically sound piece of scientific research with data that supports the conclusions. Experiments must have been conducted rigorously, with appropriate controls, replication, and sample sizes. The conclusions must be drawn appropriately based on the data presented.

Reviewer #1: Yes

Reviewer #2: Partly

Reviewer #3: Yes**

**2. Has the statistical analysis been performed appropriately and rigorously?

Reviewer #1: Yes

Reviewer #2: Yes

Reviewer #3: No**

**3. Have the authors made all data underlying the findings in their manuscript fully available?

The** [**PLOS Data policy**](http://www.plosone.org/static/policies.action#sharing) **requires authors to make all data underlying the findings described in their manuscript fully available without restriction, with rare exception (please refer to the Data Availability Statement in the manuscript PDF file). The data should be provided as part of the manuscript or its supporting information, or deposited to a public repository. For example, in addition to summary statistics, the data points behind means, medians and variance measures should be available. If there are restrictions on publicly sharing data—e.g. participant privacy or use of data from a third party—those must be specified.

Reviewer #1: Yes

Reviewer #2: Yes

Reviewer #3: Yes**

**4. Is the manuscript presented in an intelligible fashion and written in standard English?

PLOS ONE does not copyedit accepted manuscripts, so the language in submitted articles must be clear, correct, and unambiguous. Any typographical or grammatical errors should be corrected at revision, so please note any specific errors here.

Reviewer #1: Yes

Reviewer #2: Yes

Reviewer #3: Yes**

**5. Review Comments to the Author

Please use the space provided to explain your answers to the questions above. You may also include additional comments for the author, including concerns about dual publication, research ethics, or publication ethics. (Please upload your review as an attachment if it exceeds 20,000 characters)

Reviewer #1: The manuscript of Carlsson et al., PONE-D-20-11081 (Reduction of oxidative stress on DNA and RNA in obese patients after Roux-en-Y gastric bypass surgery-an observational cohort study of changes in urinary markers,) describes an interesting study which deserves to be publication. However there are several shortcomings and unclear parts which should be improved.**

Thank you for your feedback. We appreciate your suggestions in order to improve the manuscript. We will address them one by one below.

**General Remarks:
1. The description of the study is difficult to understand and a schematic illustration should be included which shows the design of the study (with exclusion ad inclusion criteria).**

In the methods section, the section about the research population has been rewritten and a schematic illustration that shows inclusion and exclusion criteria has been included in the supplemental material.

**2. Table 1 is not clear. It is mentioned that 356 patients participated at the study but when we looked at the subgroups the overall number is only 319. Furthermore, completely different numbers are mentioned in running text. The authors should check this very carefully.**

From the reviewer comments, we understand that we have not been clear enough about the fact that all patients were not categorizable in the diabetes subgroups. In former Table 1, we tried to describe the research population in full. It was explained, in the legend, that “*All patients also include patients for whom we were not able to confirm diabetes status.” And “†All patients with diabetes also include patients without hyperglycemia after RYGB, but who continued antidiabetic medicine.”

In order to clarify this, in Materials and methods section about Diabetes subgroups, we have added a new figure (Figure 1. Subdivision according to diabetes status.), illustrating the division in subgroups. In addition to this, to explain the other numbers mentioned in the running text (we assume that you hereby mean the numbers on samples at different timepoints?), a new table (Table 1. Adherence of the 356 included patients to post-operative follow up (example for 8-oxoGuo)), has been added in the Research population section. Former Table 1 has been replaced by a smaller and simpler table, only including the three large subgroups based on diabetes status (Table 2. Preoperative characteristics for the three main study population subgroups) and a revised version of former Table 1 has been moved to the supplemental materials.

**3. It is hard to believe that blood pressure values of obese people are quite low (in the normal range) before the surgery. Did the patients receive the blood lowering medications? The authors should comment these findings.**

Just as you propose, many of the patients in this population did receive anti-hypertensive treatment. We have added this information in former Table 1 (now supplemental table 1) and have added following comment in the Results section:

“all patient groups had a mean blood pressure within the normal range (consistent with an overall high prevalence of anti-hypertensive treatment).”

**4. It would be interesting that authors present in details data between individual weight-loss and reduction of base oxidation in form of graphs.**

The largest reductions in both urinary markers were seen in patients with a moderate weight loss.

We have included an illustration of this in a supplemental figure (Supplemental figure 2. Individual changes in BMI and urinary markers 24 months after RYGB) and have added following sentence to Results, section about correlations to other variables:

“The largest reductions in both urinary markers were seen in patients with a moderate weight loss, as illustrated in Supplemental figure 2, where individual patient changes in 8-oxodG and 8-oxoGuo 24 months after RYGB are shown in relation to relative postoperative BMI.”

**5. It is unclear if urinary excretion (24h) was also studied postoperatively.**

Estimates of 24-hour urinary excretion of 8-oxodG and 8-oxoGuo were also studied postoperatively. We have added “– for all pre- and postoperative samples” to the following sentence in the Materials and methods section about the physiological model: “we used a physiological model to calculate estimates of 24-hour excretion of the urinary markers – an approach that has been described in detail and validated in a recent study (42) – for all pre- and postoperative samples.”

**6. It is in general unclear which patients were monitored and which patients dropped-out. This should be clarified in a table.**

As this was a study in a “real” clinical setting, patients were monitored when they came to their medical follow up consultations. Not all patients showed up at all postoperative consultations. A new table (Table 1. Adherence of the 356 included patients to post-operative follow up (example for 8-oxoGuo)), has been added in the Research population section, as an attempt to clarify the matter of concern. In this table, it is visualized that 35 patients “dropped out” after 3 months, 48 after 6 months.

In addition to this, the following sentence has been added in the Materials and methods section: “Twelve months or later after RYGB, follow up excretion estimates could be calculated for 273 (76 %) of the included patients.”

**7. The authors mentioned differences between males and females (preoperative). The reason should be discussed.**

In our original first paper on 8-oxodG (Loft S et al. Carcinogenesis 13(1992) 2241-47) we found a difference between males and females and attributed this to female hormones. How the difference interacts with outcomes is presently not examined to greater details, and the known difference has mainly been used for confounder control, as in the present study. Thus, the levels of 8-oxodG and 8-oxoGuo differ between males and females, but the importance for outcomes is not known in much details. Therefore, we included this observation in the present paper.

In the revised version of the manuscript, the following sentences have been included in the Discussion:

“Between female and male subjects, large differences in excretion levels of both urinary markers were observed in this study. Although gender has been recognized for long as a confounding factor for 8-oxodG (44), details of the physiological background of the these differences are not known. It is also mainly unknown, and merit further studies, if the gender differences in levels of 8-oxodG and 8-oxoGuo are of importance for health outcomes.”

**The authors found also a transient increase of base oxidation after the surgery, also this finding should be.**

Surgery and particularly gastric by-pass surgery results in major stress on the body and brings about major changes in physiology, food intake and its response, healing processes, sleep – to mention a few. Other major stress situations such as extreme prolonged exercise (Poulsen et al. J Sports Sci 14(1996) 343-46); Larsen EL; Falkenberg MK et al, J Am Assoc Lab Anim Sci 58(2019) 380-389) are known to increase oxidative stress and 8-oxodG and 8-oxoGuo.

We have included the following sentence in the revision:

“We observed a transient increase in 8-oxodG after surgery. Surgery itself is a stressful event with many physiological responses. We cannot decipher specific causes for the increased oxidative stress measures after surgery, but expect that several factors are in play, and that they take some time to disappear after surgery and physiological resetting and reduction of food intake.”

**8. Discussion, Line 346: As the difference was after the adjustment not significant the authors cannot say that they confirmed early findings of an association.**

Thank you for being observant of this. We have replaced the mentioned sentence with the following:

“In our obese study population, we could not confirm the presence of a similar association after adjusting for differences in weight, height and kidney function – the variables that were included in the physiological model for estimating 24-hour excretion.”

**9. Introduction, line 65: Further studies with patients of bariatric surgery are missing in the reference list (for example Bankoglu et al., Mutagenesis 2018, 33, 61-67 and Bankoglu et al. Scientific Reports, 2018, 8)**

We have rewritten the section and have added the two suggested references. **Specific remarks:
Line 70: replace has by was**

This has been corrected

**Line 81: correlation with weight changes**

The section including this line has been rewritten

**Line 136: analyses**

This has been corrected

**Line 137: 310 random samples evaluated in 2015-2016 were repeated**

This has been corrected

**Line 142: analyses**

This has been corrected

**Line 146: 8-oxodG and 8-oxoGuo were normalized.**

This has been corrected

**Line 147: The Jaffe-method assay reference is missing.**

A reference to Jaffe’s original publication (in german) has been included.

**154: in this study, the reasons are explained in the section below.**

This has been corrected

**Line 247: triglycerides**

This has been corrected

**Reviewer #2: The manuscript investigated the impact of a weight loss in obese patients undergoing Roux-en-Y gastric bypass surgery on reduction of oxidative stress on DNA and RNA evaluating changes in urinary markers: 8-oxo-7,8-dihydro-2´-deoxyguanosine (8-oxodG) and 8-oxo-7,8-dihydroguanosine (8-oxoGuo). This work shows interesting findings, hawever I have some sugesstion:**

We are grateful. Below, we will describe, what we have done in order to improve the manuscript, based on your suggestions.

**1. the authors should briefly describe the study group**

In Materials and methods, the description of the research population has been modified:

“The research population is a cohort of patients, mainly of Caucasian ethnicity, who had surgery for obesity with the RYGB procedure between November 2010 and September 2013 and attended pre-examinations and postoperative medical follow-up at Copenhagen University Hospital Hvidovre, Denmark (33).”

Further characteristics of the specific study group are mentioned later in the manuscript, both in the section about diabetes subgroups and in Results, preoperative characteristics. Let us know if the description is not satisfactory.

**2. the authors should focus on specifically describe the patient inclusion criteria for the study, not only the exclusion criteria eg. in line 85-86 is information that ALL patients had surgery for obesity with the RYGB procedure (...); in line 87-88: Patients operated with other types of bariatric surgery, like sleeve gastrectomy or a gastric banding procedure were excluded - this this sentence is superfluous, because above the authors wrote ALL patients had RYGB**

Thank you for pointing this out, the section about inclusion and exclusion was not very clearly written.

Now, the mentioned superfluous sentence has been removed and the whole section has been revised. We have also included a schematic illustration of inclusion and exclusion criteria in the supplemental material.

**3. statistical analysis needs improvement, using a paired t Student test and calculating multiplicity adjusted p-value is more correct for 168 patients (patients who have samples before bariatric surgery and at all time intervals after surgery)**

The statistics have been revisited and we have included a repeated samples ANOVA, with planned contrasts, to show changes in time after surgery.

Only 69 patients had delivered samples at all timepoints after surgery. The number 168 is the total number of patients that had delivered samples 24 months after surgery. We have included a new table (Table 1. Adherence of the 356 included patients to post-operative follow up (example for 8-oxoGuo)) to clarify the somewhat confusing numbers in the text.

**4. in the discussion, the authors describe the results generally, but nevertheless there is no information on what might have affected the results. It is known that adipose tissue is an endocrine organ that produces adipokines affects the body's metabolism. Authors should try to explain oxidative stress influence on metabolic changes in obese patients.**

The reasons why metabolic diseases or the obese condition leads to increased formation of 8oxodG and 8oxoGuo are not known. In isolated obesity without complications, we previously reported that only 8oxoGuo is increased (15), indicating that obesity by itself increases oxidative stress by an unknown mechanism. We suggest that the obese condition is a metabolic stress to the mitochondria and that several physiological responses to obesity and overeating, for instance the release of adipokines, together make the mitochondrial respiratory chain slightly less efficient – not in the context of energy production, but in the context of increased production of ROS such as the superoxide anion, hydrogen peroxide and the hydroxy radical, that in turn will oxidize the RNAs in the vicinity of the mitochondria. RNA will in this situation function as a “photographic plate” for mitochondrial ROS production. Also, oxidized RNAs have been shown to have aberrant functions (7) with implications for disease processes (5) .

We have included the section above in the Discussion.

**5. in 358: please explain the relationship between ROS formation and DNA and RNA oxidative damage**

We have added the following lines:

“Although the exact source of ROS that oxidize DNA is not known in detail, it is believed, partly to be overlapping and partly, to some extent also to differ from the source of ROS that oxidize RNA. Although RNA and DNA oxidation are correlated, their prognostic values differ in type 2 diabetes, consistent with the view that they represent different mechanisms. The exact mechanisms need to be explored in future studies.”

**6. 8-oxo-7,8-dihydro-2´-deoxyguanosine (8-oxodG) and 8-oxo-7,8-dihydroguanosine (8-oxoGuo) are urinary markers, the authors should more explain changes in metabolic diseases**

The reasons why metabolic diseases or the obese condition lead to increased formation of 8oxodG and 8oxoGuo are not known. We have included this in the answer to comment 4. **Reviewer #3: Dear Authors,

I have read your manuscript with the great interest. The experimental design is good and in my opinion the work suits the objectives of PLOS ONE. Nevertheless, I would like to address a few comments:**

Thank you very much for your time spent revising our manuscript. In the following, we will do our best to address all the comments, one by one. **Introduction

The introduction should clearly state which biomarker is an indicator of DNA and which of RNA oxidative stress, what is the mechanism behind the difference. I suggest to move some information on this subject form the discussion section to introduction, so in the introduction the reader has whole background and in the discussion the authors only comment their results against results of others and do not explain the basic information.**

We moved some of the information on the subject from the discussion to introduction and have clarified the description of the biomarkers.

**Additionally, if the order of sentences in the last paragraph was inverted, it would create better background to state aim of the study which is lacking in this version of the manuscript.**

The last paragraph of the introduction has been completely revised and the aims have been described.

**If the aims are clearly stated, the material and methods section and results could be more structured and focused only on these elements that are essential for this paper. Please keep and follow the same sequence of the information in each section (Introduction (aims) – Materials and methods – Results – Discussion).**

We have tried to structure the sequence of information throughout the manuscript according to your suggestion.

**Please rephrase the phrase: “longitudal changes and cross-sectional differences”. Although it may be intuitive to understand it as “long-term changes” and “differences depending on gender and heatlh status”, it should be clearly stated and explained.**

This has been rephrased. **Materials and Methods

The section focuses too much on exclusion criteria and therefore is too long for the purpose of this study. I understand that the author aimed to give the best possible description of the materials and methods applied, but the amount of information makes the most important information to disappear. Please provide the initial number of patients, then provide inclusion criteria and finally give the final number of analyzed cases. All additional data might be presented in supplementary materials.**

The section about the research population has had a makeover and we have included a supplemental figure with an illustration of the inclusion and exclusion criteria.

**Also, if this is important for this study, please state why you took into account and analyzed separately the gender, the smoking status and antidiabetic treatment – it would be good to give reason for such differentiation (and how it is linked to aims of the study).**

This has now been stated in Materials and methods, statistics section.

**A scheme of the study groups would be helpful.**

An illustration (Figure 1. Subdivision according to diabetes status) has now been included as a help to the reader. **Results
My major concern is connected with statistical analysis and results presentation. Distribution of normality was not checked. If a comparison of the same parameter was made at different time points, then it should be used ANOVA for the samples associated with Mauchly's sphericity test. Table 2 shows the results of the comparisons. In this situation, two-way analysis of variance with contrast analysis should be used instead of post-hoc testing.**

A major revision has been made to the statistics in general and the statistics section in Materials and methods has been completely rewritten. We have included a repeated samples ANOVA, test of sphericity and planned contrasts. Contrast analysis has also replaced post-hoc testing in comparisons between subgroups.

In former table 2, data were not from post-hoc testing, but simple comparisons with unpaired t-tests. This has been clarified in a statement in the legend to the new table 3.  **The results are presented on the way that is very difficult to analyze it for the readers, please consider the presentation of some data in the figures, and rearrange the tables.**

Results and tables have been rearranged and figures have been revised. **Please move Table 1 to supplementary materials and leave in this section only the results that are essential for the purpose of this study and linked to the aims of this paper.**

Former table 1 has been replaced by a smaller and simpler table, only including the three large subgroups based on diabetes status (Table 2. Preoperative characteristics for the three main study population subgroups) and a revised version of former table 1 has been moved to supplemental materials.

**Please visualize data form section about bodyweight, BMI, glucose and lipid markers after RYGB.**

We have included a new figure with relative changes in body weight, BMI and markers of glucose metabolism after RYGB**.

The quality of Figure 1 is very poor, thus I cannot really read the details. I would suggest not including the results to the Figures captions: Exact p-values are reported down to 0.001. Below 0.001, p-values are reported as <0.001 or <0.0001.**

Former figure 1 has been divided in two separate figures, quality has been improved and p-values have been removed, replaced by signs marking significance after Bonferroni corrections.
 **Discussion
Please add references to previous studies quoted in lines 367-369 and elaborate on it.**

These lines are merely summarizing what has been discussed and elaborated on earlier in discussion section. As we see it, references are therefore not necessary at this point.
 **Summary:
The paper comprehensively analyses levels of DNA and RNA oxidative stress markers in urine samples of patients after RYGB. The authors made great effort to analyze substantial amount of data and correctly process them. In general, the paper is decently written, but it would be good to add clearly declared aims of the study and build the structure of the paper around it, and so in each section the information appears in the same order. This would make the paper more coherent. Also, the paper would benefit from moving some information to supplementary materials. Thank you for having the opportunity to review it.**

Your comments and suggestions have been truly valuable. We believe that the amendments made have improved the manuscript a great deal.
 **Final recommendation:
Resubmit after major revision**

A major revision has been made. We hope you find the new version satisfactory.

On behalf of the authors

Elin Rebecka Carlsson, MD
