# Supplementary material for: Reduction of oxidative stress on DNA and RNA in obese patients after Roux-en-Y gastric bypass surgery—An observational cohort study of changes in urinary markers
Source: PLoS One. 2020 Dec 14;15(12):e0243918. doi: 10.1371/journal.pone.0243918 (PMC7735613; doi:10.1371/journal.pone.0243918)
Supplement: Attachment — Submitted filename: Responce to Reviewers.docx [file pone.0243918.s006.docx]

Responce to Reviewers

**PONE-D-20-11081R1
Reduction of oxidative stress on DNA and RNA in obese patients after Roux-en-Y gastric bypass surgery – an observational cohort study of changes in urinary markers
PLOS ONE

Dear Dr. Carlsson,

Thank you for submitting your manuscript to PLOS ONE. After careful consideration, we feel that it has merit but does not fully meet PLOS ONE’s publication criteria as it currently stands. Therefore, we invite you to submit a revised version of the manuscript that addresses the points raised during the review process.

Please submit your revised manuscript by Nov 21 2020 11:59PM. If you will need more time than this to complete your revisions, please reply to this message or contact the journal office at** [**plosone@plos.org**](mailto:plosone@plos.org)**. When you're ready to submit your revision, log on to** [**https://www.editorialmanager.com/pone/**](https://www.editorialmanager.com/pone/) **and select the 'Submissions Needing Revision' folder to locate your manuscript file.

Please include the following items when submitting your revised manuscript:**

- **A rebuttal letter that responds to each point raised by the academic editor and reviewer(s). You should upload this letter as a separate file labeled 'Response to Reviewers'.**
- **A marked-up copy of your manuscript that highlights changes made to the original version. You should upload this as a separate file labeled 'Revised Manuscript with Track Changes'.**
- **An unmarked version of your revised paper without tracked changes. You should upload this as a separate file labeled 'Manuscript'.**

**If you would like to make changes to your financial disclosure, please include your updated statement in your cover letter. Guidelines for resubmitting your figure files are available below the reviewer comments at the end of this letter.

If applicable, we recommend that you deposit your laboratory protocols in protocols.io to enhance the reproducibility of your results. Protocols.io assigns your protocol its own identifier (DOI) so that it can be cited independently in the future. For instructions see:** [**http://journals.plos.org/plosone/s/submission-guidelines#loc-laboratory-protocols**](http://journals.plos.org/plosone/s/submission-guidelines#loc-laboratory-protocols) **We look forward to receiving your revised manuscript.

Kind regards,

Yvonne Böttcher, Ph.D.
Academic Editor
PLOS ONE**

**Reviewers' comments:

1. If the authors have adequately addressed your comments raised in a previous round of review and you feel that this manuscript is now acceptable for publication, you may indicate that here to bypass the “Comments to the Author” section, enter your conflict of interest statement in the “Confidential to Editor” section, and submit your "Accept" recommendation.**

**Reviewer #2: (No Response)**

**Reviewer #3: All comments have been addressed**

**2. Is the manuscript technically sound, and do the data support the conclusions?

The manuscript must describe a technically sound piece of scientific research with data that supports the conclusions. Experiments must have been conducted rigorously, with appropriate controls, replication, and sample sizes. The conclusions must be drawn appropriately based on the data presented.**

**Reviewer #2: Partly**

**Reviewer #3: Yes**

**3. Has the statistical analysis been performed appropriately and rigorously?**

**Reviewer #2: I Don't Know**

**Reviewer #3: Yes**

**4. Have the authors made all data underlying the findings in their manuscript fully available?

The** [**PLOS Data policy**](http://www.plosone.org/static/policies.action#sharing) **requires authors to make all data underlying the findings described in their manuscript fully available without restriction, with rare exception (please refer to the Data Availability Statement in the manuscript PDF file). The data should be provided as part of the manuscript or its supporting information, or deposited to a public repository. For example, in addition to summary statistics, the data points behind means, medians and variance measures should be available. If there are restrictions on publicly sharing data—e.g. participant privacy or use of data from a third party—those must be specified.**

**Reviewer #2: Yes**

**Reviewer #3: Yes**

**5. Is the manuscript presented in an intelligible fashion and written in standard English?

PLOS ONE does not copyedit accepted manuscripts, so the language in submitted articles must be clear, correct, and unambiguous. Any typographical or grammatical errors should be corrected at revision, so please note any specific errors here.**

**Reviewer #2: Yes**

**Reviewer #3: Yes**

**6. Review Comments to the Author

Please use the space provided to explain your answers to the questions above. You may also include additional comments for the author, including concerns about dual publication, research ethics, or publication ethics. (Please upload your review as an attachment if it exceeds 20,000 characters)**

**Reviewer #2: The manuscript "Reduction of oxidative stress on DNA and RNA in obese patients after Roux-en-Y gastric bypass surgery - an observational cohort study of changes in urinary markers" is written correctly, but there are some remarks:**

Thank you again for your time and for your comments. In the following, we have tried to address the concerns one by one and hope that you will find the adjustments satisfactory.

**1.in the abstract and the main text, the term "short term" should be replaced with a specific time interval, e.g. 1 week / month after bariatric surgery**

Line 37: “In the short term after RYGB” has been replaced with “in the first months after RYGB”.

Line 42-44: The conclusion-paragraph in abstract has been replaced with another, as suggested in 5.

Line 376: “short term” has been replaced with “the first months”

Line 447: The sentence has been rephrased.

**2. in line 84 the phrase "similar to sleeve gastrectomy" should be deleted**

The phrase “similar to sleeve gastrectomy” has been deleted.

**3. in line 88 and 89: "The studies investigating one or several different markers of oxidative stress and antioxidant defense after RYGB in obese patients show conflicting results (25–31)" - the sentence should be briefly expanded, what results were obtained?**

The sentence has been expanded:

“Most of the studies investigating one or several different markers of oxidative stress and antioxidant defense after RYGB in obese patients suggest an improvement of oxidative stress after the surgery, although results are partly conflicting – for instance some of the studied markers (glutathione, superoxide dismutase and catalase) have been reported to be increased after RYGB in some of the studies and to be decreased in other studies (25–31).”

**4. in research population, the criteria for including / excluding patients from the study should be described clearly (comorbidities, autoimmune and viral diseases, medications taken, dietary supplements, antioxidants)**

In the section about the research population, we have added the following section, describing the inclusion criteria for Roux-en-Y gastric bypass surgery:

“The criteria for inclusion were a BMI above 35 together with one or several comorbidities related to obesity (type 2 diabetes, resistant hypertension, sleep apnea, infertility in women with polycystic ovary syndrome, lower extremity arthrosis), or a BMI above 50, without complications. Minimum age was 25 years. Exclusion criteria were, in addition to general contraindications for surgery and/or anesthesia, severe psychiatric disorder, eating disorder or substance abuse. All patients were instructed to take vitamin and mineral supplementations according to international nutritional recommendations and common practice (34). Unable- or unwillingness to follow the nutritional recommendations was an exclusion-criteria.

**5. the conclusion should be described in more detail**

The conclusion in abstract (lines 42-44) has been rephrased to contain more of the details in summary:

“We conclude, that oxidative damage to nucleic acids, reflected in the excretion of 8-oxodG and 8-oxoGuo, had decreased significantly two years after RYGB – indicating that reduced oxidative stress could be contributing to the many long-term benefits of RYGB-surgery in obesity and type 2 diabetes.”

**Reviewer #3: Dear Authors,

I recommend the current version of the revised manuscript to be published in PLOS ONE.
Congratulations of interesting study and manuscript.**

Thank you so much, it has been a pleasure working with you on this revision.

On behalf of the authors

Elin Rebecka Carlsson, MD
